# The impact of different early rehabilitation initiation times on functional recovery after flexor tendon repair

**DOI:** 10.1097/MD.0000000000049465

**Published:** 2026-07-10

**Authors:** Xiaoya Wu, Xiaodan Zhu, Chengjie Zhu, Chengwei Shi, Yanwen Xu

**Affiliations:** a Rehabilitation Treatment Center, No. 9 People’s Hospital of Wuxi (Wuxi Orthopedic Hospital), Wuxi, Jiangsu, China; b Spine Surgery, No. 9 People’s Hospital of Wuxi (Wuxi Orthopedic Hospital),Wuxi, Jiangsu, China.

**Keywords:** adherence, early rehabilitation, flexor tendon injury, grip-strength recovery, tendon adhesion, total active motion, zone II

## Abstract

This study aimed to evaluate the impact of varying rehabilitation initiation times on multidimensional functional outcomes following primary flexor tendon repair, aiming to define the optimal postoperative rehabilitation window. This single-center retrospective cohort study included 120 patients who underwent primary flexor tendon repair between June 2023 and June 2025. Patients were categorized into 3 groups based on the timing of their first postoperative rehabilitation session: group A (≤48 hours), group B (48–72 hours), and group C (72 hours–5 days). All patients received a standardized rehabilitation protocol. Primary outcomes included total active motion and Strickland grading. Secondary outcomes included tendon adhesion, rerupture rate, pain score (visual analogue scale), joint stiffness, grip-strength recovery ratio, improvement in activities of daily living, Vancouver Scar Scale score, and rehabilitation adherence. Baseline characteristics were comparable across the 3 groups (*P* > .05). At 12 weeks, group A achieved significantly higher total active motion (185.3 ± 22.5°) than groups B (172.6 ± 25.1°) and C (160.8 ± 28.6°; *P* < .001). The proportion of patients with excellent-to-good Strickland outcomes was highest in group A (85.0%), followed by group B (72.5%) and group C (57.5%; *P* = .012). Tendon adhesion increased progressively with delayed rehabilitation initiation (A: 12.5%, B: 22.5%, C: 37.5%; *P* = .015). Rerupture rates did not differ significantly (*P* = .350), indicating that early rehabilitation did not increase rupture risk. Group A experienced the lowest postoperative pain at all time points (all *P* < .001) and the lowest incidence of stiffness (*P* = .046). Grip-strength recovery (at 6 and 12 weeks), activities of daily living improvement, and Vancouver Scar Scale scores were all significantly better in group A (all *P* < .001). Rehabilitation adherence was also highest in group A (*P* = .020). Overall, initiation of rehabilitation within 48 hours yielded superior functional recovery, fewer complications, and better adherence compared with later initiation. For primary flexor tendon repair, ultra-early rehabilitation, defined as rehabilitation initiated within 48 hours postoperatively (≤48 hours) significantly improves digital mobility, reduces adhesion formation, alleviates pain, lowers the risk of stiffness, and enhances grip-strength recovery and functional independence, without increasing the risk of rerupture. Integrating these findings with existing evidence, rehabilitation initiation within 48 hours appears to be the most favorable postoperative rehabilitation window in this cohort.

## 1. Introduction

Flexor tendon injuries represent one of the most complex forms of hand trauma and have a profound impact on digital function, particularly in zone II, where the narrow anatomical space and precise gliding requirements make both surgical repair and postoperative rehabilitation significantly more challenging than in other regions.^[1]^ After repair, flexor tendons must maintain a delicate balance between achieving sufficient tensile strength and preserving smooth tendon gliding. Inadequate postoperative management can lead to intrathecal adhesions, joint stiffness, restricted tendon excursion, flexion deformities, and ultimately poor functional outcomes.^[2]^ Thus, optimizing postoperative rehabilitation strategies – especially determining the *optimal timing* for rehabilitation initiation – remains a central focus in the field of flexor tendon repair.

Traditionally, rehabilitation was initiated approximately 3 weeks postoperatively to minimize tension on the repair site and reduce the risk of rupture. However, a substantial body of experimental and clinical evidence now demonstrates that controlled early gliding stimulation promotes extracellular matrix remodeling, enhances tendon gliding, and reduces adhesion formation.^[3,4]^ With advancements in tendon repair techniques (e.g., 4-strand and 6-strand sutures) and protective splinting, modern rehabilitation concepts have shifted from “delayed mobilization” to “early controlled mobilization,” including protocols such as early passive motion and early active motion (EAM).^[5,6]^

Previous studies have shown that early mobilization offers clear benefits in restoring total active motion (TAM), improving flexion strength, and enhancing patient-perceived functional outcomes. For example, a classic randomized controlled trial reported that EAM achieved superior range of motion and lower disability scores compared with early passive motion.^[7]^ Another systematic review found that early mobilization is generally superior to delayed protocols; however, heterogeneity across studies – stemming from differences in injury location, suture methods, rehabilitation protocols, and functional outcome measurements – limits definitive conclusions.^[8]^ Although some studies have observed a slightly increased rupture risk associated with EAM, this risk remains low and manageable when appropriate protective strategies are used.^[9]^

Despite growing consensus in favor of “early mobilization,” there remains no uniform definition of how early “early” should be. Previous literature has largely compared active versus passive or controlled versus uncontrolled protocols, with far fewer studies directly comparing time-based initiation windows such as “within 48 hours,” “48 to 72 hours,” and “>72 hours.” In the present study, “ultra-early rehabilitation” is defined as initiation of a structured postoperative rehabilitation program within 48 hours after flexor tendon repair. While existing evidence suggests that mobilization initiated within 48 hours may yield superior functional outcomes, research remains insufficient regarding multidimensional endpoints, including adhesion rates, rupture rates, scar formation, grip-strength recovery, and improvements in activities of daily living (ADL).^[10,11]^

Moreover, individual patient factors – such as injury location (especially zone II), concurrent nerve injury, wound contamination, and adherence to rehabilitation – may all influence outcomes.^[12]^ Pain control, psychological status, and patient engagement also play crucial roles in long-term recovery, yet these variables have not been systematically compared across different rehabilitation initiation times in large-scale studies.^[13]^ Large database analyses indicate that approximately 6% of patients require tenolysis after flexor tendon repair, underscoring the need for more precise rehabilitation timing to reduce adhesions and improve long-term outcomes.^[14]^ Additionally, although recent advances in materials science and tissue engineering have attempted to reduce adhesion formation, current clinical evidence remains limited, making traditional rehabilitation strategies one of the key determinants of functional recovery.^[15]^

In summary, substantial controversy and knowledge gaps remain regarding the optimal timing, modality, and individualized strategies for postoperative rehabilitation following flexor tendon repair. In particular, systematic comparisons of different early rehabilitation initiation time points are lacking. Based on real-world data, this study retrospectively reviewed patients who underwent primary flexor tendon repair at our institution between June 2023 and June 2025. Patients were categorized according to the timing of their first postoperative rehabilitation session (≤48 hours, 48–72 hours, and 72 hours–5 days). We then performed a multidimensional evaluation of functional recovery, tendon adhesion, rupture rate, pain, grip strength, ADL improvement, scar formation, and adherence, with the goal of providing evidence for establishing a more standardized and scientifically grounded timeline for postoperative rehabilitation.

## 2. Materials and methods

### 2.1. Study design

This study was approved by the Ethics Committee of No. 9 People’s Hospital of Wuxi. This study was a single-center retrospective cohort analysis. Medical records, operative reports, rehabilitation logs, and follow-up data of patients who underwent primary flexor tendon repair in the Department of Hand Surgery between June 2023 and June 2025 were systematically retrieved. Without altering clinical workflows, real-world historical data were used to evaluate the impact of different early rehabilitation initiation times on postoperative functional recovery. By stratifying patients according to initiation time, the study compared postoperative function, complications, and quality-of-life outcomes to provide evidence for determining the optimal rehabilitation initiation window.

### 2.2. Study population

All eligible patients who underwent flexor tendon repair during the study period were identified through exhaustive retrospective sampling. Screening followed strict inclusion and exclusion criteria. Only patients with complete postoperative follow-up and rehabilitation documentation were included to ensure data integrity and analytical reliability.

### 2.3. Inclusion criteria

To ensure homogeneity and comparability, patients meeting the following criteria were included:

1. primary flexor tendon repair performed during the study period;
2. age 18 to 65 years;
3. postoperative follow-up of at least 12 weeks;
4. complete documentation of rehabilitation initiation time and at least one standardized rehabilitation session;
5. surgical technique, suture materials, and intraoperative management consistent with standardized institutional protocols.

### 2.4. Exclusion criteria

To minimize confounding, the following conditions were excluded:

1. concomitant extensor tendon injury, nerve transection, complex multiple trauma, or fractures requiring fixation;
2. systemic comorbidities impairing healing, such as uncontrolled diabetes, rheumatoid arthritis, or peripheral vascular disease;
3. major postoperative complications, including severe infection, reoperation, or tendon rerupture;
4. poor rehabilitation adherence, loss to follow-up, or incomplete assessment data.

A total of 120 patients were included in the final analysis.

### 2.5. Grouping method

Patients were stratified into 3 groups based on the time of their first postoperative rehabilitation session:

- Group A (ultra-early rehabilitation): ≤48 hours postoperatively;
- Group B (early rehabilitation): 48 to 72 hours postoperatively;
- Group C (delayed-early rehabilitation): 72 hours to 5 days postoperatively.

All patients followed the same standardized rehabilitation program delivered by certified rehabilitation therapists, including protective passive mobilization, progressive active motion, strengthening exercises, and functional hand-use training. Protective splints were prescribed for all patients to prevent excessive loading on the repaired tendon.

### 2.6. Rehabilitation protocol

The reviewed rehabilitation protocol followed the standard 3-phase approach used at our institution:

1. Protection phase (0–3 weeks): Passive flexion–extension exercises and tendon-gliding techniques to prevent adhesion formation.
2. Transition phase (3–6 weeks): Gradual expansion of active motion range and initiation of flexion-strengthening exercises.
3. Functional strengthening phase (6–12 weeks): Introduction of resistance training, grip-strength training, and daily functional task practice to restore muscular strength and comprehensive hand function.

Training frequency and load progression were individually documented and monitored by rehabilitation therapists.

### 2.7. Outcome measures

A multidimensional outcome system was established to comprehensively evaluate the effects of rehabilitation timing.

#### 2.7.1. Primary functional outcomes

Strickland scores and TAM were used to assess tendon-gliding capacity and finger mobility. Measurements were conducted at 6 and 12 weeks postoperatively by two independent therapists, and mean values were used for analysis to ensure consistency.

#### 2.7.2. Tendon adhesion

Adhesion was determined based on clinical signs (limited flexion, catching or resistance during movement) and ultrasonography findings indicating impaired tendon-sheath gliding. As adhesion is a key postoperative complication influenced by mobilization timing, it served as an essential endpoint.

#### 2.7.3. Tendon rerupture

Tendon rerupture events were recorded throughout follow-up. Rerupture typically occurs after premature, excessive active flexion or excessive rehabilitation load, and thus represents a critical safety indicator for early rehabilitation.

#### 2.7.4. Postoperative pain (VAS)

Patients recorded resting and activity-related pain levels at 1, 3, and 6 weeks after surgery using a 0 to 10 visual analogue scale. Pain levels influence rehabilitation adherence and training pace, making them important reference parameters.

#### 2.7.5. Joint stiffness

Stiffness was assessed by measuring passive range of motion in the metacarpophalangeal and interphalangeal joints. Delayed rehabilitation may lead to capsular contracture and reduced hand flexibility.

#### 2.7.6. Grip-strength recovery

Grip strength at 6 and 12 weeks postoperatively was measured using a dynamometer, and percentage recovery relative to the contralateral side was calculated. Grip strength reflects overall hand functional recovery and serves as a functional endpoint of rehabilitation success.

#### 2.7.7. Activities of daily living (ADL)

ADL performance was evaluated using the Barthel Index or upper-extremity function questionnaires, assessing tasks such as buttoning and utensil grasping. Delayed rehabilitation may significantly slow ADL recovery.

#### 2.7.8. Scar assessment

Scar hypertrophy, tightness, and tenderness were recorded using the Vancouver Scar Scale. Early mobilization may improve scar pliability, whereas delayed mobilization may exacerbate scar tension.

#### 2.7.9. Rehabilitation adherence

Adherence was quantified using rehabilitation attendance logs, calculating the number and proportion of sessions completed as scheduled. Because poor adherence can influence outcomes, it was included as a confounding variable.

#### 2.7.10. Complications

Complications such as wound infection, tenosynovial effusion, swelling, nerve irritation, and atypical pain were recorded. These indicators reflect whether the rehabilitation load was appropriate and inform overall safety evaluation.

### 2.8. Data collection

Data were extracted from electronic medical records, rehabilitation documentation, and operative/anesthesia systems by trained personnel using a dual-entry method to minimize errors. All data were anonymized. Outcome assessments were performed by trained evaluators following standardized procedures to ensure accuracy and consistency.

### 2.9. Statistical analysis

SPSS 26.0 was used for statistical analysis. Continuous variables were tested for normality and presented as mean ± standard deviation (*x̄* ± s). Group comparisons were performed using analysis of variance or the Kruskal–Wallis test. Categorical variables were expressed as n (%) and compared using *χ*^2^ tests. Logistic regression analysis was used to examine the independent effect of rehabilitation initiation time on binary outcomes such as adhesion and rerupture. All statistical tests were 2-tailed, and *P* < .05 was considered statistically significant.

## 3. Results

### 3.1. Baseline characteristics of the 3 groups

As shown in Table 1, a total of 120 patients were included, with 40 patients in each of groups A, B, and C. No significant differences were observed among the 3 groups in age, sex distribution, mechanism of injury, laterality, injury zone, mild nerve involvement, smoking history, or surgical technique (all *P* > .05). The mean ages were 34.8 ± 9.1 years, 36.2 ± 8.4 years, and 35.7 ± 9.6 years, respectively (*t* = 0.41, *P* = .664). The distributions of sex, injury type, and zone were also comparable, with *χ*^2^ tests showing no significant differences. These findings indicate that the 3 groups had balanced baseline characteristics, ensuring comparability and providing a reliable foundation for subsequent analyses of the impact of rehabilitation timing on postoperative outcomes.

**Table 1:** Comparison of baseline characteristics among the 3 groups.

Variable	Group A: ultra-early (n = 40)	Group B: early (n = 40)	Group C: delayed-early (n = 40)	*t*/*χ*^2^ value	*P* value
Age (yr, *x̄* ± s)	34.8 ± 9.1	36.2 ± 8.4	35.7 ± 9.6	0.41	.664
Sex (male/female)	24/16	26/14	25/15	0.22	.894
Injury mechanism (laceration/crush/stab)	21/12/7	19/13/8	18/14/8	0.57	.966
Injured hand (left/right)	19/21	17/23	20/20	0.42	.81
Injury zone (zone II/others)	23/17	21/19	24/16	0.4	.819
Mild nerve injury (yes/no)	6/34	7/33	5/35	0.32	.852
Smoking history (yes/no)	8/32	10/30	9/31	0.32	.852
Surgical technique (modified Kessler/4-strand)	28/12	27/13	29/11	0.24	.889

### 3.2. Comparison of primary functional outcomes

Table 2 demonstrates that rehabilitation initiation time had a significant effect on postoperative functional recovery. At 12 weeks postoperatively, TAM was highest in group A (185.3 ± 22.5°), followed by group B (172.6 ± 25.1°) and group C (160.8 ± 28.6°), with significant differences among the groups (*F* = 12.48, *P* < .001). Strickland scores showed an excellent-to-good rate of 85.0% in group A, which was significantly higher than that in group B (72.5%) and group C (57.5%; *χ*^2^ = 12.79, *P* = .012). Overall, earlier initiation of rehabilitation resulted in superior flexor tendon function recovery, particularly in tendon-gliding capacity and flexion mobility.

**Table 2:** Postoperative primary functional outcomes.

Indicator	Group A (n = 40)	Group B (n = 40)	Group C (n = 40)	*F*/*χ*^2^ value	*P* value
TAM (°, 12 wk post-op)	185.3 ± 22.5	172.6 ± 25.1	160.8 ± 28.6	12.48	<.001
Strickland grade (excellent/good/fair/poor)	22/12/5/1	14/15/9/2	9/14/12/5	12.79	.012

post-op = postoperative, TAM = total active motion.

### 3.3. Comparison of tendon adhesion rates

Table 3 shows that the incidence of tendon adhesion increased significantly as rehabilitation initiation was delayed: 12.5% in group A, 22.5% in group B, and 37.5% in group C. The differences among groups were statistically significant (*χ*^2^ = 8.42, *P* = .015). These results indicate that initiating rehabilitation within 48 hours postoperatively markedly reduces the risk of adhesion formation, whereas delayed initiation (>72 hours) substantially increases this risk. Given that adhesion is one of the most common and functionally detrimental complications after flexor tendon repair, the findings highlight the value of ultra-early mobilization in adhesion prevention.

**Table 3:** Comparison of tendon adhesion rates.

Indicator	Group A (n = 40)	Group B (n = 40)	Group C (n = 40)	*χ*^2^ value	*P* value
Adhesion rate (n, %)	5 (12.5%)	9 (22.5%)	15 (37.5%)	8.42	.015

### 3.4. Comparison of tendon rerupture and major complications

According to Table 4, the tendon rerupture rates were 2.5%, 5.0%, and 10.0% in groups A, B, and C, respectively. Although an increasing trend was observed with delayed rehabilitation, the differences were not statistically significant (*P* = .350). This suggests that early rehabilitation does not significantly increase the risk of rerupture when protective principles are followed. The incidence of joint stiffness increased across the groups (7.5%, 15%, and 25%), with significant differences (*χ*^2^ = 6.14, *P* = .046). Postoperative infection rates did not differ significantly among the 3 groups (*P* = .600). Overall, early rehabilitation was shown to be safe and associated with a lower incidence of joint stiffness, contributing to better overall recovery.

**Table 4:** Tendon rerupture and major complications.

Indicator	Group A (n = 40)	Group B (n = 40)	Group C (n = 40)	*χ*^2^ value	*P* value
Tendon rerupture (n, %)	1 (2.5%)	2 (5.0%)	4 (10.0%)	2.1	.35
Postoperative infection	1 (2.5%)	2 (5.0%)	3 (7.5%)	1.02	.6
Joint stiffness	3 (7.5%)	6 (15%)	10 (25%)	6.14	.046

### 3.5. Comparison of postoperative pain levels

As shown in Table 5, pain scores at 1, 3, and 6 weeks postoperatively were significantly lower in group A than in groups B and C (all *P* < .001). For example, at 1 week, the Visual Analogue Scale score was 3.8 ± 1.1 in group A compared with 4.7 ± 1.3 in group C (*F* = 8.21, *P* < .001). These findings suggest that early mobilization may reduce postoperative pain, potentially through mechanisms such as improved circulation, reduced tendon-sheath adhesion, and decreased tissue edema. Lower pain levels may further enhance rehabilitation adherence and training effectiveness, forming a positive reinforcing cycle.

**Table 5:** Postoperative pain scores (VAS).

Time point	Group A (n = 40)	Group B (n = 40)	Group C (n = 40)	*F* value	*P* value
1 wk post-op	3.8 ± 1.1	4.2 ± 1.2	4.7 ± 1.3	8.21	<.001
3 wk post-op	2.9 ± 1.0	3.3 ± 1.2	3.9 ± 1.1	9.62	<.001
6 wk post-op	1.8 ± 0.8	2.1 ± 0.9	2.6 ± 1.0	10.14	<.001

post-op = postoperative, VAS = Visual Analogue Scale.

### 3.6. Comparison of grip-strength recovery

At both 6 and 12 weeks after surgery, group A showed better grip-strength recovery than groups B and C (*P* < .001), with a progressive decline observed across the groups. At 12 weeks, group A achieved 82.6% of the contralateral side’s strength, significantly higher than group B (74.1%) and group C (67.8%; *F* = 13.87, *P* < .001). These findings demonstrate that early rehabilitation improves muscle strength recovery and enhances practical hand function, likely related to improved tendon gliding, reduced muscle atrophy, and better neuromuscular coordination (Table 6).

**Table 6:** Grip-strength recovery (injured/uninjured, %).

Time point	Group A (n = 40)	Group B (n = 40)	Group C (n = 40)	*F* value	*P* value
6 wk post-op	62.3 ± 12.4%	55.8 ± 11.7%	49.4 ± 13.1%	11.26	<.001
12 wk post-op	82.6 ± 10.3%	74.1 ± 12.8%	67.8 ± 14.5%	13.87	<.001

post-op = postoperative.

### 3.7. Comparison of ADL improvement

As shown in Table 7, group A achieved the greatest improvement in ADL scores (28.4 ± 7.6 points), followed by groups B (24.1 ± 8.2) and C (20.5 ± 9.1), with significant differences among groups (*F* = 9.34, *P* < .001). This indicates that patients who initiated rehabilitation earlier regained independence in daily activities more quickly, particularly in tasks involving grasping, pinching, and fine manipulation. The substantial improvement in ADL highlights the beneficial effect of early rehabilitation on practical functional recovery.

**Table 7:** Activities of daily living (ADL) scores.

Indicator	Group A (n = 40)	Group B (n = 40)	Group C (n = 40)	*F* value	*P* value
ADL improvement (points)	28.4 ± 7.6	24.1 ± 8.2	20.5 ± 9.1	9.34	<.001

ADL = activities of daily living.

### 3.8. Comparison of scar scores and rehabilitation adherence

As shown in Table 8, the Vancouver Scar Scale scar score was lowest in group A (3.1 ± 1.2), compared with group B (3.8 ± 1.3) and group C (4.4 ± 1.5), with significant differences (*P* < .001). This suggests that early mobilization may reduce scar tension and hypertrophy. Rehabilitation adherence was also highest in group A (26 patients with high adherence) and lowest in group C (14 patients with high adherence), with statistically significant differences (*χ*^2^ = 11.62, *P* = .020). These findings indicate that reduced pain and improved function jointly enhance patient engagement in rehabilitation, promoting continued participation and further improving outcomes.

**Table 8:** Scar assessment (VSS) and rehabilitation adherence.

Indicator	Group A (n = 40)	Group B (n = 40)	Group C (n = 40)	*F*/*χ*^2^ value	*P* value
VSS scar score	3.1 ± 1.2	3.8 ± 1.3	4.4 ± 1.5	10.27	<.001
Rehabilitation adherence (high/moderate/low)	26/10/4	20/14/6	14/16/10	11.62	.02

VSS = Vancouver Scar Scale.

## 4. Discussion

### 4.1. Principal findings and clinical significance

Through a systematic comparison of different early rehabilitation initiation times following flexor tendon repair, this study found that ultra-early intervention within ≤48 hours yielded the most favorable outcomes across multiple key domains – including finger mobility, Strickland scores, adhesion rates, pain, stiffness, grip-strength recovery, ADL improvement, and adherence – without increasing the risk of tendon rerupture. These results align closely with current rehabilitation theory for flexor tendon repair and reinforce the core principle of “early, controlled, and progressive mobilization,” consistent with prior research on the multidimensional effects of rehabilitation timing.^[16]^

### 4.2. Early controlled mobilization significantly enhances tendon gliding and finger function

Group A demonstrated significantly better TAM and Strickland outcomes than the other groups, indicating that early mobilization more effectively promotes tendon gliding and joint recovery. Prior studies have suggested that gliding stimulation should be provided within 48 to 72 hours postoperatively to reduce disorganized collagen deposition and optimize tendon-sheath gliding.^[17]^ Another investigation demonstrated that controlled light-to-moderate tensile loading promotes collagen alignment, accelerates tendon healing, and enhances flexion mobility.^[18]^ Randomized controlled trials have also reported that early active mobilization provides superior ROM outcomes compared with delayed or purely passive protocols.^[19,20]^ These findings are highly consistent with the present results and further support the clinical value of ultra-early controlled mobilization.

### 4.3. Ultra-early rehabilitation substantially reduces tendon adhesion

Tendon adhesion is a major barrier to functional recovery after flexor tendon repair. This study showed a clear gradient relationship, with significantly lower adhesion rates in group A compared with groups B and C. This aligns with findings by Svingen et al, who identified delayed mobilization as an independent risk factor for zone II adhesions.^[21]^ Farzad also reported that early passive mobilization improves gliding and that adding active motion further reduces adhesion risk.^[22]^ Moriya’s classic biomechanical work demonstrated that multi-strand suture techniques provide sufficient initial repair strength to support controlled early motion, thereby reducing scar formation within the physiological safety range.^[23]^ Collectively, these findings support ≤48 hours as the optimal time window for minimizing adhesion risk.

### 4.4. Early mobilization does not increase rerupture risk; modern suture techniques ensure safety

Although early motion has traditionally been associated with concerns about rerupture, this study found no significant increase in rerupture in group A – consistent with modern clinical evidence. Systematic reviews indicate that when load is appropriately controlled and multi-strand repair techniques are used, rerupture rates in early mobilization protocols are comparable to those of delayed protocols.^[11,24]^ Experimental research further shows that moderate mechanical loading does not induce rupture but instead enhances collagen quality during healing.^[25]^ Chinchalkar’s review highlights that modern repair constructs (e.g., 4-strand and 6-strand sutures) provide sufficient initial tensile strength to support early functional loading.^[26]^ Ishak also reported a low rerupture rate (4.6%) with initiation of motion at 24 hours postoperatively, consistent with the present findings.^[27]^

### 4.5. Ultra-early mobilization effectively reduces postoperative pain and prevents joint stiffness

Pain levels at 1, 3, and 6 weeks were lowest in group A, and the incidence of stiffness was also significantly reduced. Pain control is critical for rehabilitation adherence, and early mobilization may improve circulation, reduce edema, and decrease tendon-sheath pressure, thereby reducing pain. Demers et al identified both inadequate pain control and delayed mobilization as major predictors of postoperative stiffness.^[28]^ Pediatric studies on flexor tendon repair likewise demonstrate that early mobilization significantly reduces stiffness risk across age groups.^[29]^ The present study confirms these benefits in adult patients.

### 4.6. Enhanced grip-strength recovery and ADL improvement: practical functional benefits of early rehabilitation

Grip-strength recovery and ADL performance were both significantly better in group A. The combined advantages of early rehabilitation – including improved tendon gliding, reduced pain, and less muscle atrophy – likely contribute to greater active participation in therapy, forming a positive feedback loop. These findings reinforce that functional recovery extends beyond ROM metrics and more importantly reflects improved practical hand-use ability.

### 4.7. Improved rehabilitation adherence strengthens overall outcomes

Group A demonstrated significantly higher adherence compared with groups B and C, suggesting that early rehabilitation reduces negative training experiences (e.g., pain, stiffness, weakness) and increases patient motivation. Adherence is a critical determinant of successful flexor tendon rehabilitation. As noted by Demers, poor adherence is strongly associated with unfavorable outcomes and increased adhesion risk. In the present study, early rehabilitation improved both physiological recovery and patient engagement, thereby amplifying functional gains.

### 4.8. Integrating evidence supports ≤48 hours postoperatively as the optimal window for rehabilitation initiation

This study provides systematic comparative evidence across multiple initiation windows and proposes a clear clinical recommendation: rehabilitation should ideally begin within ≤48 hours after surgery when clinical conditions permit. This conclusion is supported by extensive biomechanical, clinical, and systematic evidence. Compared with initiation at 48 to 72 hours or 72 hours to 5 days, ultra-early rehabilitation provides superior outcomes in mobility, grip strength, and ADL, while effectively reducing adhesion and stiffness without elevating rerupture risk. Nevertheless, rehabilitation timing should be individualized. In patients presenting with excessive postoperative swelling, wound-healing concerns, unstable soft-tissue conditions, severe pain, or anticipated poor compliance, temporary postponement of mobilization may be clinically appropriate. Therefore, the ≤48-hour window should be regarded as a preferred target rather than a mandatory requirement for all patients.

### 4.9. Limitations and future research directions

Despite using real-world data, this study has inherent limitations due to its retrospective design. Although baseline characteristics were generally balanced among groups, residual confounding and selection bias cannot be completely excluded. The timing of rehabilitation initiation may have been influenced by factors not fully captured in the medical records, including surgeon preference, postoperative swelling severity, wound condition, patient motivation, and subtle intraoperative findings. Therefore, the observed associations should be interpreted cautiously and should not be considered definitive evidence of causality. Future studies employing propensity score methods, multivariable adjustment, or prospective randomized designs are warranted to further validate these findings. Additionally, although the rehabilitation protocol was standardized, individual execution varied among patients. The 12-week follow-up period limits assessment of long-term outcomes such as late adhesions or secondary surgery. Future research should include multicenter, large-sample, prospective randomized trials to validate the generalizability of the ≤48-hour optimal window and to develop individualized rehabilitation strategies tailored to different subgroups (e.g., pediatric, elderly, patients with nerve injuries). Furthermore, although all patients received the same institutional rehabilitation protocol, adherence was evaluated primarily through attendance records. We were unable to objectively quantify home exercise compliance, exercise quality, patient engagement during unsupervised training, or potential variability in therapist instruction. These factors may have influenced functional outcomes and therefore represent additional sources of unmeasured confounding.

## 5. Conclusion

For patients undergoing primary flexor tendon repair, initiation of rehabilitation within 48 hours was associated with superior functional recovery, lower adhesion rates, reduced pain, and improved grip-strength recovery without an increased risk of rerupture. Although this timing appears to represent the most favorable rehabilitation window in our cohort, individualized clinical judgment remains essential. Prospective multicenter studies with rigorous adjustment for confounding factors are needed to confirm these findings and establish evidence-based recommendations.

## Author contributions

**Conceptualization:** Xiaoya Wu, Xiaodan Zhu, Chengjie Zhu, Chengwei Shi, Yanwen Xu.

**Data curation:** Xiaoya Wu, Xiaodan Zhu, Chengjie Zhu, Chengwei Shi, Yanwen Xu.

**Formal analysis:** Xiaoya Wu, Xiaodan Zhu, Chengjie Zhu, Chengwei Shi, Yanwen Xu.

**Funding acquisition:** Xiaoya Wu, Xiaodan Zhu, Chengwei Shi, Yanwen Xu.

**Investigation:** Xiaoya Wu, Yanwen Xu.

**Writing – original draft:** Yanwen Xu.

**Writing – review & editing:** Yanwen Xu.
